# Massive hemorrhage from an aortoesophageal fistula caused by esophageal stent implantation: A case report and literature review

**DOI:** 10.1097/MD.0000000000018303

**Published:** 2019-12-20

**Authors:** Yefei Zhan, Zhaojun Xu

**Affiliations:** Department of Intensive Care Unit, Hwa Mei Hospital, University of Chinese Academy Of Sciences, Ningbo, China.

**Keywords:** aortoesophageal fistula, computed tomography, endoscopy, esophageal stent, gastrointestinal bleeding

## Abstract

**Rationale:**

Aortoesophageal fistula (AEF) is the direct communication between the aorta and esophagus, which can cause fatal hemorrhage, and its incidence increased with the use of an esophageal stent (ES).

**Patient concerns:**

A 79-year-old man was admitted due to hemodynamic shock with massive hematemesis caused by AEF 1 month after the implantation of an ES.

**Diagnoses:**

Computed tomography angiography visualized an AEF with an ulcer-like projection on the aortic arch where the ES was placed. Angiography of the aorta revealed extravasation of contrast media from the aortic arch into the stented esophagus, which confirmed the diagnosis.

**Interventions:**

Thoracic endovascular aortic repair (TEVAR) was performed for massive hematemesis caused by ES-related, AEF but did not solve the underlying problem, leading to the second fatal hemorrhage.

**Lessons:**

TEVAR for the unique treatment of ES-related AEF is feasible in certain cases but may lead to collapse after a specific period.

## 1 Introduction

Aortoesophageal fistula (AEF), the direct communication between the aorta and esophagus, is a rare but life-threatening condition with an annual incidence of 0.007 per million.^[1]^ The main causes of AEF are aortic diseases, such as aneurysm (54.2%), ingestion of foreign bodies (19.2%), and advanced esophageal carcinoma (17.0%), and, less frequently, secondary causes are aortic or esophageal operations (4.8%).^[2]^ Recently, different designs of esophageal stents (ESs) have emerged to improve dysphagia and the quality of life of patients with malignant esophageal tumor, malignant fistula, or extrinsic compression. However, these stents carry a risk of adverse events, such as hemorrhage, pain, and fistula. This report describes a case of gastrointestinal bleeding caused by AEF that developed 1month after ES placement. Moreover, we provided a literature review on the current knowledge in the field.

## 2 Case presentation

A 79-year-old Chinese Han man with a history of squamous cell carcinoma of the esophagus was treated with surgical excision 3 months before admission. He denied having any family history of digestive disease.

**Figure 1:**
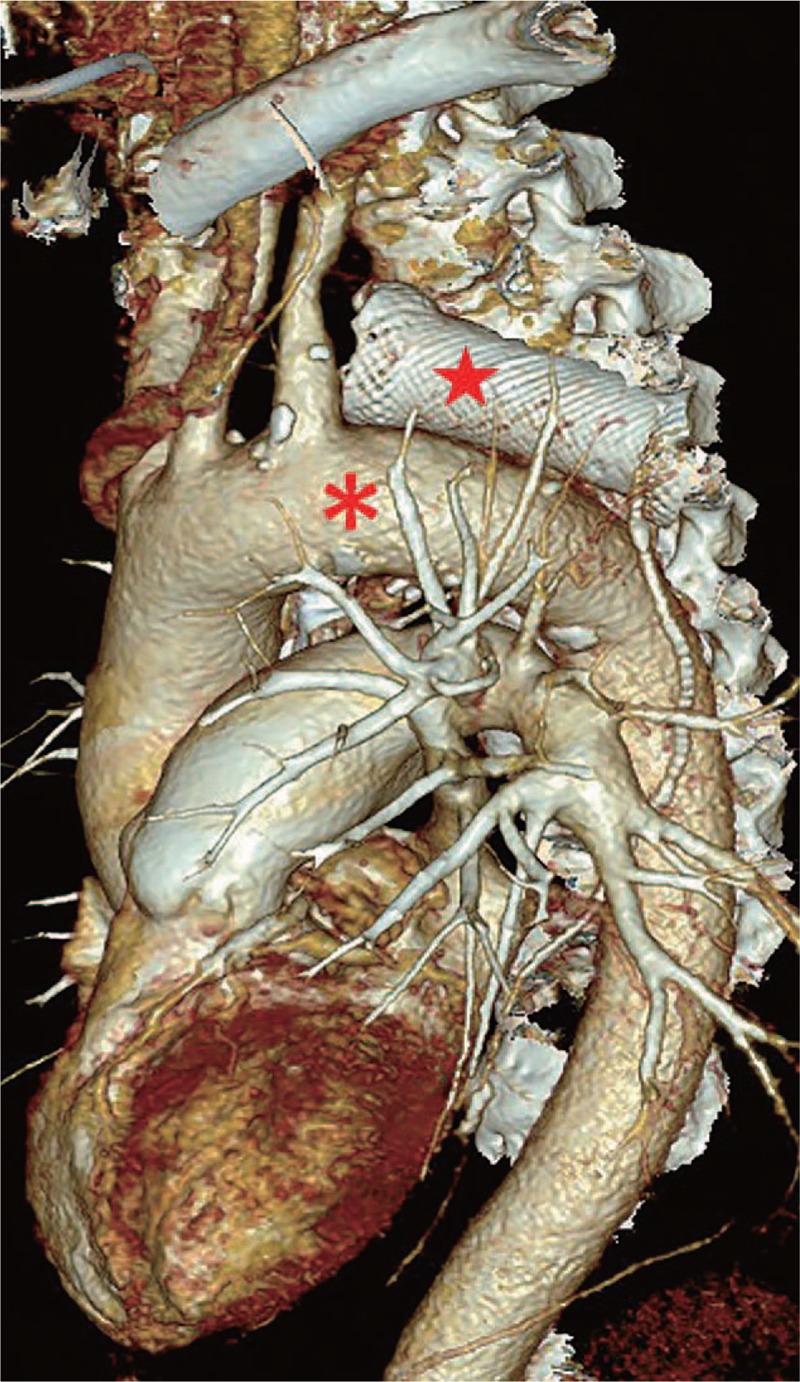
The reconstructed computed tomographic image revealed that the esophageal stent (red star) is placed close above the aortic arch (red asterisk).

**Figure 2:**
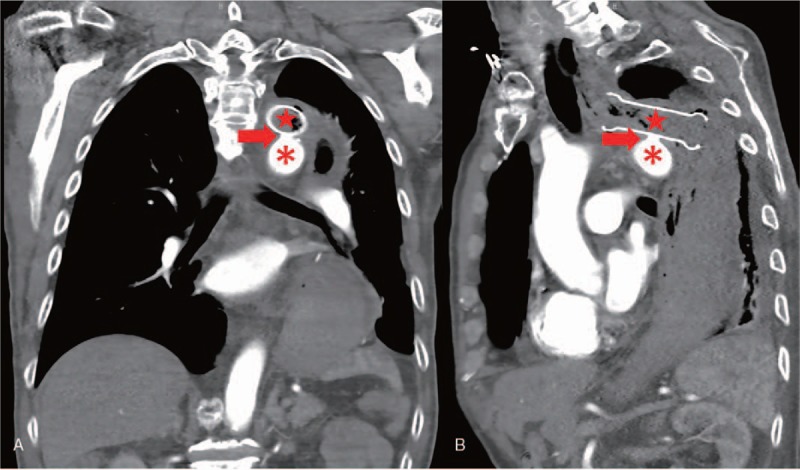
Computed tomographic angiography visualized an aortoesophageal fistula with an ulcer-like projection (red arrow head) on the aortic arch (red asterisk) where the esophageal stent (red star) touched. (A) Coronal position. (B) Sagittal position.

**Figure 3:**
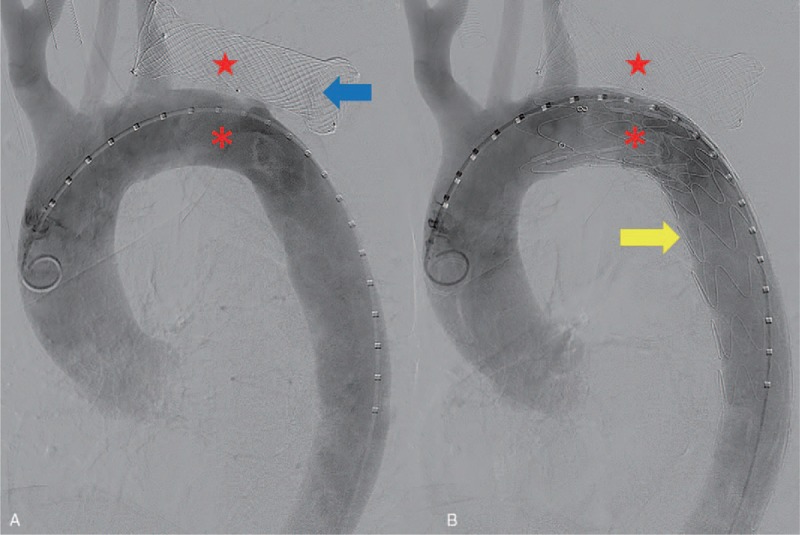
Angiography of the aorta revealed extravasation of contrast media (blue arrow head) from the aorta (red asterisk) into the stented esophagus (A, red star). The thoracic endovascular stent graft (yellow arrow head) placed on the aortic arch to rescue the case of massive hematemesis caused by an aortoesophageal fistula (B).

He presented with progressive dysphagia and esophageal metallic stent placement (20 × 70 mm, the upper edge was 24 cm from the incisors) for anastomotic stenosis (27.5 cm from the incisors) 1 month before. At this time, he was transferred to our emergency department due to hematemesis and tarry stool. Abdominal computed tomography (CT) revealed only postoperative changes in esophageal cancer and esophageal metallic stent, which was placed close above the aortic arch (Fig. 1). Upon arrival at the intensive care unit, the patient vomited approximately 500 mL of fresh blood. On physical examination, he was oriented and diaphoretic with a pale conjunctiva. He had no heart murmur, clear breathing sounds, and an old operative scar over the left chest, and the abdomen was soft without tenderness. Blood pressure (BP) was 72/56 mmHg, heart rate was 108 beats/min, respiratory rate was 28 breaths/min, and oxygen saturation was 91% in room air. Hemoglobin level was 6.8 g/dL. Large volumes of blood products were rapidly transfused, including 7.5 units of packed cells and 340 mL of fresh frozen plasma, and noradrenaline were administered simultaneously. When the patient's BP reached 90/60 mmHg, gastroscopy demonstrated large quantities of fresh blood and blood clots in the esophagus and stomach, so the source of the bleeding could not be identified. A vascular rupture was highly suspected. CT angiography (CTA) of the aorta was performed, which disclosed an aortic arch with possibly localized rupture (Fig. 2). A cardiovascular surgeon performed an emergent angiography of the aorta, which showed bleeding from the wall of the aortic arch, so a diagnosis of AEF was confirmed, and an long covered stent (26 × 200 mm) was implanted (Fig. 3) for thoracic endovascular aortic repair (TEVAR). The patient underwent fasting and received antibiotic treatment (intravenous cefodizime [2.0 g] once every 12 hours for 2 weeks and then replaced with intravenous combination of levofloxacin [0.5 g] once a day and piperacillin-tazobactam [4.5 g] once every 12 hours for another 2 weeks). The patient recovered well and was admitted to the department of gastroenterology on post-admission day 2. On post-admission day 9, esophagogastroduodenoscopy showed neither endoleakage nor peptic ulcer, and a jejunal feeding tube was established to provide enteral nutrition. Re-hematemesis occurred abruptly and massively on post-admission day 26, and the patient eventually died. The son of the patient provided informed consent for the publication of the case.

## 3 Discussion

The first report on AEF was in 1818 due to a beef bone fragment.^[3]^ Chiari^[4]^ first described AEF in 1914 as a triad of midthoracic pain or dysphagia followed by sentinel hemorrhage and fatal exsanguination after a symptom-free interval of hours to days. AEFs are more common (68%) than aortobronchial fistulas (5%), and both types of fistulas coexist in 26% of cases.^[5]^

In 1983, Frimberger^[6]^ treated a patient diagnosed with an esophageal stricture with stent placement. ESs became increasingly more popular for patients with dysphagia^[7,8]^ and are also used for hemostasis by stenting during severe esophageal bleeding caused by acute necrotizing esophagitis and acute esophageal variceal bleeding.^[9–11]^ A previous study reported that, of 153 patients with ES-related adverse events, 43 died, accounting for 28.1% of all adverse events. Of these, 14 deaths were caused by massive bleeding, accounting for 32.6% of all deaths and 9.15% of all adverse events.^[12]^ Aryaie et al^[13]^ retrospectively reported using ES to treat 20 patients with anastomotic leaks after foregut surgery. Of these, the treatment in 2 patients (10%) was complicated by AEF formation. Attention should be paid to ES-related AEF. Reports in the literature are presented in Table 1.^[13–23]^ The time of ES-related AEF development varies greatly from 18 days to 11 months after ES implantation. The causes of ES-related AEF are as follows: injury, tearing, or rupture due to repeated mechanical actions for interventional operations; high pressure from the ES to the esophageal wall affecting the blood supply of the nourishing vessels of the esophagus or increased swelling at both ends of the ES causing localized ischemia, necrosis, or ulceration resulting in AEF; tumor growth and invasion; and placement of the ES at an angle with the esophageal wall rubbing between ES and esophageal wall with vessel pulsation and respiratory movement, leading to AEF.^[24]^ The main risk factors for the development of ES-related AEF were previous repeated dilations, previous radiotherapy, proximal stricture location, and inappropriate stent choice.^[14]^ Shortened retrievable ES placement time or use of biodegradable fully covered ESs could reduce the incidence of AEF.

**Table 1:**
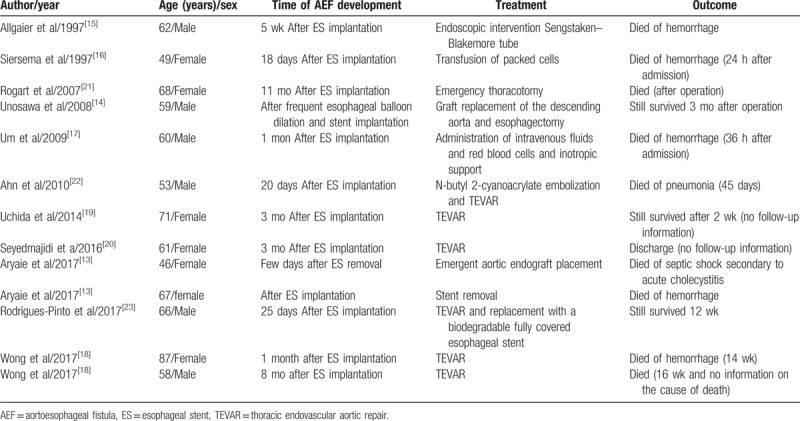
Case series of AEF after esophageal stent implantation.

CTA can reveal the size and location of an aorta, so it is a good diagnostic tool for ES-related AEF with a sensitivity of 40% to 90% and specificity of 33% to 100%.^[25]^ In this report, AEF was further specified by angiography of the aorta, which is considered as the “diagnostic criterion standard.”

Compared with only conservative treatment, the patients who underwent TEVAR survived for much more days than patients who did not underwent treatment for rupture of the aorta, who died hours after hematemesis.^[13,15–17]^ The reported mortality rate of AEF is nearly 77% with intervention and 100% without treatment.^[26]^ Clinically conservative management includes the use of broad-spectrum antibiotics and proton pump inhibitors and potential enteral feeding via percutaneous endoscopic gastrostomy or esophageal fistula bypass, but outcomes are always fatal due to recurrent hemorrhage or chronic infection and mediastinitis.^[5]^ Serious efforts have been made to improve the long-term survival rate of AEF since the first patient to survive after surgical treatment was reported in 1983.^[27]^ Since 1994, when endovascular treatment to manage aortic lesions of AEF was first reported,^[28]^ TEVAR has become a rapid, less invasive, and effective alternative to surgical intervention for the urgent and emergent management of patients with AEF. It provides rapid hemodynamic stabilization by controlling bleeding from the fistula site.^[24,18]^ However, different causes have different prognoses. AEF caused by foreign body ingestion^[29–31]^ after TEVAR treatment has better prognosis than ES-related AEF.

This case indicates that TEVAR for the unique treatment of ES-related AEF is feasible in the certain cases,^[19,20]^ but may lead to collapse after a specific period.^[32,33]^ Failure to treat the underlying cause will result in poor outcomes. The first cause of aggravation is that TEVAR does not treat the esophageal defect, which is a source of infection, and thus increases the risk of rehemorrhage, mediastinitis, sepsis, and death, with a poor long-term prognosis. The second cause is that direct friction between the ES and aorta still exists after TEVAR. Thus, surgery is needed for cleaning the infected lesion or friction as further treatment, which means definitive management requires open procedures.^[34,35]^ The control of the etiology of AEF is considered the Achilles’ heel of treatment. An autopsy was not performed in the current case because the patient's family would not grant consent. This is the limitation of this case report.

## Author contributions

**Conceptualization:** Zhaojun Xu.

**Formal analysis:** Yefei Zhan.

**Funding acquisition:** Yefei Zhan.

**Investigation:** Yefei Zhan.

**Supervision:** Zhaojun Xu.

**Writing – original draft:** Yefei Zhan.

**Writing – review & editing:** Zhaojun Xu.
